# Meta-analysis of association between the Pro12Ala polymorphism of the peroxisome proliferator–activated receptor-γ2 gene and diabetic retinopathy in Caucasians and Asians

**Published:** 2012-09-10

**Authors:** Jinlan Ma, Yan Li, Fang Zhou, Xiaoyi Xu, Gang Guo, Yi Qu

**Affiliations:** 1 Department of Ophthalmology, Qilu Hospital of Shandong University, Jinan, China; 2 Department of Ophthalmology, Shandong Provincal Hospital, Jinan, China; 3 Qilu Hospital of Shandong University, Jinan, China; 4 Department of Health Care, Qilu Hospital of Shandong University, Jinan, China

## Abstract

**Purpose:**

The Pro12Ala polymorphism of the peroxisome proliferator–activated receptor-γ2 (*PPARγ2*) gene is reported to be associated with diabetes. However, the gene’s association with diabetic retinopathy (DR) in type 2 diabetes mellitus (T2DM) has been investigated in numerous epidemiologic studies with controversial results. This meta-analysis aimed to collectively assess the association of the Pro12Ala polymorphism with DR in T2DM.

**Methods:**

An electronic literature search was conducted on PubMed, ISI Web of Knowledge, EMBASE, and the China National Knowledge Internet. A dominant model [(Pro/Ala +Ala/Ala) versus Pro/Pro] was used to ensure adequate statistical power. Crude odds ratios (ORs) and 95% confidence intervals (CIs) were calculated using the fixed effect model. Potential sources of heterogeneity and bias were explored.

**Results:**

This meta-analysis included genotype data from 2,720 cases with DR and 2,450 controls free of DR from eight eligible publications. The results showed the Ala allele had a protective effect on DR in T2DM (OR=0.81; 95% CI: 0.68–0.98, p=0.03). There was no significant evidence against homogeneity (I^2^=46%, P_heterogeneity_=0.07). The sensitivity analysis showed a robust association of the Pro12Ala polymorphism with DR in T2DM after a study involving Caucasians that presented a big effect on heterogeneity (OR=0.75; 95% CI: 0.62–0.91, p=0.003) was excluded. Possible ethnic differences in the association of the Pro12Ala single nucleotide polymorphism and DR were demonstrated; a significant association was illustrated in the Caucasian subgroup (OR=0.74; 95% CI: 0.59–0.94, p=0.01) but was not found in the Asian subgroup (OR=0.77; 95% CI: 0.55–1.07, p=0.12). No publication bias was observed.

**Conclusions:**

This meta-analysis suggested a significant association exists between the Pro12Ala polymorphism and DR in T2DM with ethnic differences. The Ala allele had a significant protective effect against DR in T2DM.

## Introduction

Diabetic retinopathy (DR) is the fifth most common cause of irreversible vision loss in working-age adults in the world, accounting for approximately 4.8% of global blindness [1]. Over time, almost all diabetic individuals eventually develop DR.

Epidemiologic studies suggest that the severity of DR closely correlates with the glycemic level and diabetes duration [2,3]. Mounting evidence indicates a significant genetic contribution to the severity of DR as well [4-6]. One putative genetic determinant of DR in type 2 diabetes mellitus (T2DM) is the Pro12Ala polymorphism in the gene encoding peroxisome proliferator–activated receptor γ *(PPARγ*) [7]. *PPARγ* is a nuclear transcription factor involved in adipocyte differentiation, glucose and lipid metabolism, and fatty acid transport. A more common Pro12→Ala substitution in the *PPARγ* gene was detected in several ethnic groups [8]. Researchers reported that the Pro12Ala single nucleotide polymorphism (SNP) plays a key role in regulating the expression of numerous genes involved in lipid metabolism, metabolic syndrome, inflammation, and atherosclerosis [9,10]. Moreover, several studies demonstrated that the SNP Pro12Ala was associated with greater insulin sensitivity [11,12], lower body mass index (BMI) [13], and diabetes [12,14]. Once diabetes has developed, the protective effect of the Ala allele may be lost. Then vascular complications increase, and more β-cell dysfunction is observed [15,16].

The frequency of the Ala allele of the Pro12Ala polymorphism in the *PPAR-γ2* gene has been reported to range from 2% to 18% in healthy people [17]. The allele is most commonly detected in Caucasians (12%) and comparatively low in Asians (4% of Japanese and 1% of Chinese) [16,18]. The effect of this rare allele on an individual is weak; however, the population-attributable protection is enormous [17].

Recently, some reports demonstrated that the Ala allele is associated with reduced risk of diabetic nephropathy [19-21] in T2DM, whereas controversial outcomes were demonstrated for DR [22-26], even though many biochemical, genetics, and functional studies have strongly indicated that the *PPARγ* gene may be sensitive for DR [27,28]. Understanding of the role of the Pro12Ala polymorphism in DR may accelerate the development of novel pharmacological agents to prevent or treat DR and related disorders. The meta-analysis performed in this study aimed to identify the relationship between the Pro12Ala polymorphism in the *PPARγ2* gene and DR of T2DM. Furthermore, the potential of the Pro12Ala polymorphism as a candidate genetic therapeutic marker in patients with DR is discussed.

## Methods

### Search strategy

**Figure 1:**
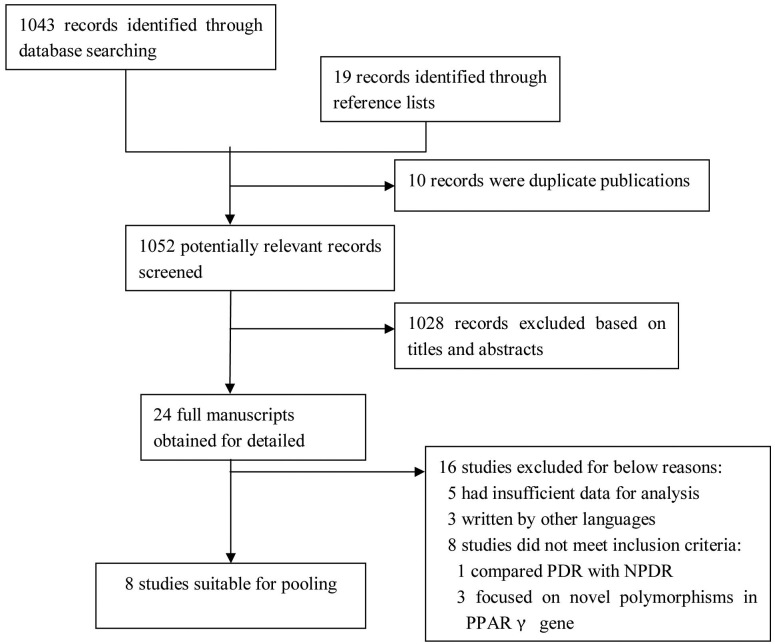
Detailed process of the literature selection. All relevant articles were evaluated by two investigators based on the title, keywords, and abstract at first, then full texts of all retrieved publications were reviewed and identified by inclusion criteria.

An electronic search was conducted for relevant available articles published in English and Chinese in four databases: the PubMed database (National Center for Biotechnology Information, NCBI), ISI Web of Knowledge (Version 4.5), EMBASE, and the China National Knowledge Internet. The search used the following keyword strings: “gene,” “diabetic retinopathy,” “PPARγ2,” “diabetic complications,” “peroxisome proliferator–activated receptor,” “T2DM,” and “type 2 diabetes mellitus,” and was limited by “humans,” “clinical trial,” “adult,” and time before November 2011. Meanwhile, other studies were collected via a manual search. Publications that appeared twice or focused on other diabetic complications based on the same study group were removed. All relevant articles identified through the search were scanned based on the title, keywords, and abstract (where available) by at least two investigators and were rejected in the initial screening if the article clearly did not meet the inclusion criteria. Where a title/abstract could not be rejected with certainty, full texts of all retrieved publications were reviewed and evaluated. The reference list of each relevant publication was also examined to identify additional studies appropriate for inclusion in the meta-analysis. The literature selection process is shown in Figure 1.

### Inclusion criteria

Studies were selected independently by two interviewers according to the following inclusion criteria [1]: a case–control or cohort study published as an original study evaluating the association of the Pro12Ala polymorphism in *PPARγ2* with the risk of DR in T2DM [2]; ophthalmological examinations diagnosing DR with ophthalmoscopy after pupillary dilatation [3]; numbers in case and control groups or exposed and unexposed groups reported for each genotype, or data provided from which numbers could be calculated [4]; and case and control groups in a case–control study or exposed and unexposed groups in a cohort study unrelated and drawn from the same temporally and geographically defined underlying population. If the two investigators disagreed about the eligibility of an article, the disagreement was resolved by consensus with a third reviewer.

### Exclusion criteria

The following exclusion criteria were used: articles written in languages other than English or Chinese [2]; review articles, case reports, and abstracts [3]; missing genotype-specific case numbers or number of patients with DR [4]; and missing deviation measurements.

### Data extraction

The following information was extracted from published reports with a standardized protocol and reporting form: the number of cases (subjects with T2DM and DR) and controls (subjects with T2DM without DR) and subjects’ genotype information, such as allele or genotype frequencies, the first author’s last name, year of publication, study design, ethnicity, subject characteristics at baseline, including age, sex, BMI, smoking status, history of diabetes, hypertension, diabetes complications, total cholesterol, and glycated hemoglobin.

### Statistical analysis

The Pro12Ala genotypes include Pro/Pro, Pro/Ala, and Ala/Ala. The frequencies of the minor homozygous genotype Ala/Ala were low, and we used a dominant model [(Pro/Ala +Ala/Ala) versus Pro/Pro] for the primary meta-analysis to ensure adequate statistical power.

The subjects in this study were from different geographical areas, and each subpopulation was treated as a separate comparison. The subgroup analyses were defined as Caucasians and Asians.

Data were processed by RevMan (Version 5.0; The Cochrane Collaboration, Copenhagen, Denmark). The distribution of genotypes was checked for the Hardy–Weinberg equilibrium (HWE). The HWE of each SNP in the control group of each study was examined by using χ2 analysis; studies not in the HWE were subjected to a sensitivity analysis. The between-study heterogeneity was tested with the chi-square-based Cochran’s statistic and the inconsistency index (I^2^). Statistically significant heterogeneity was considered present with P_heterogeneity_<0.05 and I^2^>50%. In the presence of substantial heterogeneity (I^2^>50%), the random effect model (REM) was adopted as the pooling method; otherwise, when I^2^<50%, the fixed effect model (FEM) was used as the pooling method. The leave one out sensitivity analysis was performed using I^2^ >50% as the criterion for evaluating the key studies with a substantial impact on between-study heterogeneity. The odds ratio (OR) of the Ala allele in DR was calculated with the Mantel–Haenszel test in FEM or the DerSimonian & Liard test in REM; p<0.05 is considered nominally significant. Meta-regression with restricted maximum likelihood estimation was performed to assess the potentially important covariates exerting a substantial impact on between-study heterogeneity. A funnel plot was performed to look for evidence of publication bias. The funnel plot should be asymmetric when there is publication bias or symmetric in the case of no publication bias. Beggar’s and Egger’s tests were used to find publication bias.

## Results

### Characteristics of studies

After a thorough literature search, eight eligible publications including 2,450 cases and 2,720 controls were involved in this meta-analysis according to the inclusion criteria, including six studies of Caucasian patients [20,23-25,29] and two studies of Asian patients [16,19]. All studies provided data about the participants with DR (including non-proliferative DR and proliferative DR) as cases and controls without DR in T2DM. Table 1 summarizes the characteristics of the included studies. All subjects’ ages on average ranged from 59.2 to 70.7 years, the duration of T2DM on average ranged from 9.1 to 19.5 years, and gender was not evenly distributed. Further efforts were conducted on the subgroup analyses according to ethnicity defined as Caucasians and Asians. The genotype distribution in the control group was consistent with the HWE (Table 1).

**Table 1:** Characteristics of involved studies

**Included studies**	**Ethnicities**	**Number of cases/controls**	**Age (years)**	**BMI (kg/m^2^)**	**Duration of T2DM (years)**	**Sex (male)**	**HWE (P-value)**
[29]	Caucasian	100/106	64.2±8.4*	34.2±3.71*	16.5±6.4*	96*	0.157
[20]	Caucasian	69/376	59.2±10*	28.1±4.8*	10*	220*	0.42
[23]	Caucasian	88/136	NA	31.4±6.2/31.1±6.1	15.1±7.7/9.1±6.8	52/109	0.984
[24]	Caucasian	160/101	66.7±9.1/70.7± 9.0	27.8±4.5/27.7±4.4	19.5±8.8/16.4±6.9	71/41	0.537
[25]	Caucasian	100/403	59.8±10.5*	27.8±5.0*	11.7±8.2*	253*	0.327
[26]	Caucasian	196/319	66.2±9.3*	27.4±3.9*	9.6±7.9*	318*	0.525
[19]	Asian	382/378	64.37±11.2*	NA	10.9*	NA	0.241
[16]	Asian	1626/575	60.9±11.7*	23.2±3.6*	NA	995*	0.817

Data are n, means±SD (n), or % (n), some data are given by Case/Control, some data could only be extracted by total,*data are given in total. NA, data are not available; BMI, Body Mass Index; T2DM, Type 2 Diabetes Mellitus; HWE, Hardy–Weinberg equilibrium.

### Statistics summary

The prevalence of the Ala allele was 10.28% and 19.47% in the cases and controls, respectively. Polymorphism frequencies varied by ethnicity; the frequency of the Ala allele in the controls was observed to be greater in Caucasian populations (27.09%) than in Asian populations (7.56%).

**Figure 2:**
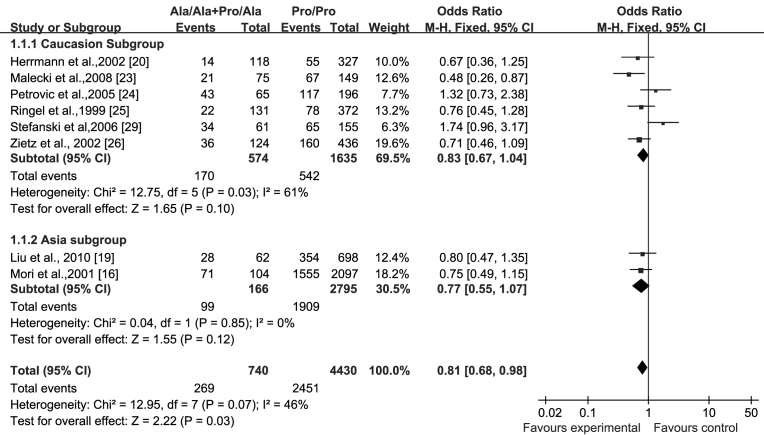
Forest plots of association of Pro12Ala polymorphism with diabetic retinopathy. The forest plot compared genotypes Ala (Ala/Ala+Pro/Ala) with Pro/Pro in Caucasian and Asian subgroups. In all eight studies, patients with diabetic retinopathy were treated as cases, and patients with diabetes without retinopathy were treated as controls. Squares indicate the study-specific odds ratio (OR); the size of the box is proportional to the weight of the study. Horizontal lines indicate 95% confidence intervals (CIs). Diamonds indicate summary ORs with corresponding 95% CIs. Our report indicated the statistical difference of incidence of diabetic retinopathy happened in Ala carriers compared with Pro/Pro carriers in total, and no significant association was found in Caucasian and Asian subgroups.

According to the forest plot (Figure 2), we adopted the FEM in this analysis based on the overall I^2^<50%. Eight studies examining the relationship of the Pro12Ala polymorphism and DR in T2DM in the dominant model yielded a summary OR of 0.81 (95% CI: 0.68–0.98, p=0.03), which means the incidence of DR was lower in subjects with the Ala variant than in those without it. No significant association was found in the Caucasian and Asian subgroups, respectively; the FEM OR was 0.83 (95% CI: 0.67–1.04, p=0.10) in Caucasians and 0.77 (95% CI: 0.55–1.07, p=0.12) in Asians. In the sensitivity analysis, after Stefanski’s (2006) study [29] excluded, the relationship between the Pro12Ala polymorphism and DR was robust in the remaining seven studies, yielding a summary OR of 0.75 (95% CI: 0.62–0.91, p=0.003). Moreover, the result was positive in the Caucasian subgroup (OR=0.74; 95% CI: 0.58–0.94, p=0.01; Table 2).

**Table 2:** Pooled measurements on the relationship of Pro12Ala polymorphism in the *PPARγ2* gene with diabetic retinopathy

**Groups**	**Before/after sensitivity analysis**	**Articles included**	**Numbers of cases/controls**	**FEM pooled OR (95% CI)**	**P**	**I^2^(%)**	**Pheterogeneity**
All relevant articles	Overall analysis	8	2720/2450	0.81 (0.68–0.98)	0.03	46	0.07
	Sensitivity analysis	7	2621/2333	0.75 (0.62–0.91)	0.003	1	0.42
Caucasians	Overall analysis	6	712/1497	0.83 (0.67–1.04)	0.1	61	0.03
	Sensitivity Analysis	5	613/1380	0.74 (0.58–0.94)	0.01	33	0.2
Asians	Overall analysis	2	2108/953	0.77 (0.55–1.07)	0.12	0	0.85

All results based on dominant model, Ala/Ala+Pro/Ala VS Pro/Pro; CI, conﬁdence interval; OR, odds ratio; I^2^, inconsistency index; FEM, fixed effect model. Significant difference of incidence of diabetic retinopathy was found in Ala carriers comparing with Pro/Pro carriers in Caucasians.

### Heterogeneity and sensitivity analysis

The overall I^2^ in this study was 46% (P_heterogeneity_=0.07), which means the heterogeneity among the eight studies is acceptable. Between-study heterogeneity in Caucasians (I^2^=61%, P_heterogeneity_=0.03) was greater than in Asians (I^2^=0, P_heterogeneity_=0.85), which led to the leave one out sensitivity analysis (via excluding the studies one by one) to explore the potential sources of between-study heterogeneity. After Stefanski’s (2006) [29] study was excluded, the heterogeneity almost disappeared (I^2^=1%, P_heterogeneity_=0.42), which indicated that this study can be identified as the main contributor of heterogeneity. We reevaluated Stefanski’s (2006) [29] study in terms of design, statistics and methodology, selection bias, publication bias, citation bias, and multiple publication bias and did not find anything wrong.

### Publication bias evaluation

**Figure 3:**
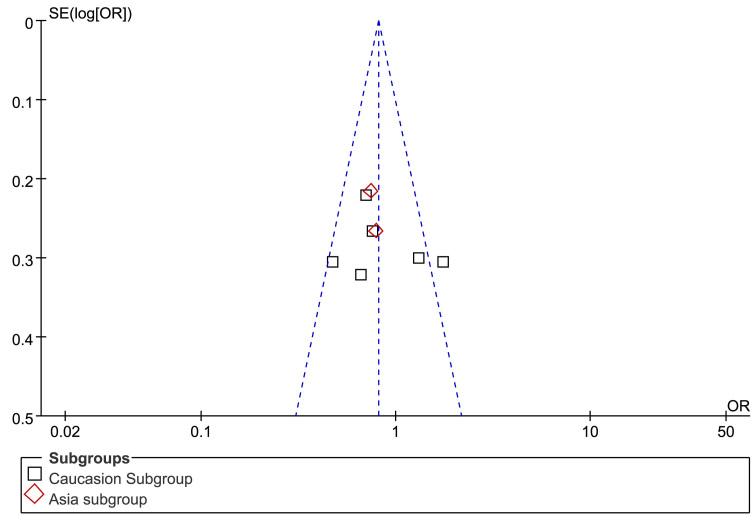
Funnel plot of the evaluation of publication bias in the association between the Pro12Ala polymorphism and diabetic retinopathy in all eight studies. The funnel plot should be asymmetric when publication bias exists. No significant publication bias was found in this meta-analysis.

The funnel plot was symmetric because no significant publication bias was found within the eight studies (Figure 3). Consistent results were drawn from the Beggar and Egger’s tests.

## Discussion

This meta-analysis yielded evidence that the Pro12Ala polymorphism of the *PPAR*γ gene is associated with DR in T2DM. The Ala allele of the Pro12Ala polymorphism carried a protective effect against DR in T2DM patients. The *PPARγ* gene plays a key role in glucose metabolism, angiogenesis, and inflammation pathways, and the growing evidence of the anti-inflammatory, oxidative, and proliferative effects of the gene’s synthetic and natural ligands strongly suggest that this nuclear receptor is a primary target in DR treatment. The gene was also considered a potential candidate gene of DR development [22,23].

Numerous studies have investigated the effects of the *PPARγ*2 gene on DR, but the results were contradictory and inconclusive [22-25]. The lack of concordance across these studies reflected limitations such as small sample sizes, age, sex, difference in ethnicity, and research methodology. However, as a complex disease, DR results from a complicated interplay of genetic and environmental factors, and the contributing pathogenetic role of the Pro12Ala polymorphism in the *PPARγ* gene in cooperation with other factors should be elucidated. Therefore, this meta-analysis obtained a more definitive conclusion regarding the role of the Pro12Ala polymorphism in the risk of DR.

Our findings were based on eight gene-association studies, which involved 5,170 participants and were robust in terms of all the planned and performed sensitivity analyses. In the eight studies, two, Stefanski (2006) [29] and Petrovic (2005) [24], demonstrated a trend toward higher incidence of DR in Ala allele carriers; however, the others [16,19,20,23,25] indicated a lower risk of developing DR in Ala carriers. We calculated the OR with the Mantel-Haenszel test and noticed that the Pro12Ala SNP was marginally associated with DR in T2DM, and a protective effect of Ala allele in the incidence of DR existed. Regarding ethnic differences, we performed the subgroup analyses defined by Caucasians and Asians, but no significant association was present between the Pro12Ala SNP and DR in different ethnicities. Perhaps the bigger heterogeneity of Caucasians are responsible (I^2^=61%), which could cover the true outcome.

Furthermore, we performed a sensitivity analysis to observe the source of heterogeneity and found Stefanski’s (2006) study [29] played a crucial role in conducting heterogeneity. After this study was excluded, the robust association of the Pro12Ala SNP with DR was detected in the seven remaining studies; moreover, the five remaining Caucasian studies yielded positive results in the subanalyses, whereas, the subanalyses were negative in Asian populations. This critical finding illustrates the significant association of the Pro12Ala SNP with DR. We also found an ethnic difference among different ethnic groups. The CIs in the Caucasian populations did not intersect with the vertical line at 0, indicating the results were statistically significant at the 0.05 level. The CIs in the Asian populations intersected with the vertical line at 0, meaning there were no statistical differences. This result was consistent with Radha et al.’s study [30], which reported the Ala allele did not protect South Asian populations against T2DM but did protect Caucasians. The present meta-analysis corroborated previous studies by suggesting that the Ala allele is a protective factor against DR in Caucasian populations. There are three possible reasons for the differences among ethnicities: First, the Ala allele is most commonly detected in Caucasians (12%) but is comparatively low in Asians (4% of Japanese and 1% of Chinese). Second, a large proportion of heterogeneity between Asians and Caucasians can be explained by the BMI; the protective effect of the Ala allele was greater in participants with lower BMIs [31]. Luan et al. hypothesized a gene-nutrient interaction based on the ratio of polyunsaturated fats to saturated fats that determined the association between the Pro12Ala polymorphism and BMI [32]. These studies [31,32] may contribute toward an explanation of the role played by ethnic differences in dietary habits. In addition, we also assumed that the waist-to-hip ratio and the duration of follow-up could account for heterogeneity among different ethnicities. Third, only two Asian studies were found in the search; thus, we cannot exclude that the lack of association of Pro12Ala and DR in Asians might be due to the limited number of studies and the consequent lack of statistical power. More studies are needed in the future.

Indeterminate numbers of characteristics that vary among studies could be the cause of between-study heterogeneity. A potential source of variation was identified by Radha et al. [30]. Differences in the association of the Pro12Ala SNP with T2DM between men and women were reported, and the relationship between the Pro12Ala polymorphism and T2DM in men was absent in women. Population stratification, design quality, non-comparable measure of genotyping, variation of the covariate, etc., can increase the heterogeneity in genome-wide association studies in complex diseases; they should be taken into account as a source of heterogeneity. We conducted a meta-regression to describe the reasons for heterogeneity, which showed none of the covariates mentioned above had a significant impact on between-study heterogeneity.

The mechanism behind the effects of the Pro12Ala polymorphism on DR has not yet been investigated in detail. A study [33] reported that *PPARγ2* Ala allele carriers had higher BMI and fat-mass but not a worse metabolic profile, possibly because of a more favorable adipose tissue distribution. The main location of *PPARγ* expression is adipose tissue, which influences the lipid metabolism and adipocyte differentiation. The adipocytes produce hormones, cytokines, and free fatty acids. All of these factors may cause structural and functional dysfunction of the retina vasculature.

Our report combined multiple studies from different subpopulations, increased generalizability, and overall strength, and helped to overcome the prior inconsistencies in the literature.

The current meta-analysis has several limitations that may affect the conclusions. First, the *PPARγ2* gene has been shown to be associated with BMI, total cholesterol, and diabetic nephropathy. A report conducted a meta-analysis [34] on the association of these factors with the Pro12Ala SNP; therefore, we did not perform the same analyses. Second, several SNPs in the human *PPARγ2* gene have been identified; however, we selected only the Pro12Ala polymorphism because this polymorphism was the most extensively studied. Meta-analyses that investigate the association of other polymorphisms in the *PPARγ*2 gene with DR should be performed in the future. Third, it was difficult to get full papers published in various languages; we included only studies published in English and Chinese.

In conclusion, this meta-analysis identified the significant relationship between the Pro12Ala polymorphism in the *PPARγ2* gene and DR in T2DM. The Ala carriers have a lower chance of developing DR than the Pro allele carriers, so we can draw the hypothesis that the Ala allele of the Pro12Ala polymorphism in the *PPARγ2* gene has a protective effect in the incidence of DR in patients with T2DM. We also found ethnic differences existed, in which the Ala allele demonstrated protection in Caucasians but not in Asians. Further detailed explorations are required to detect the role of the *PPARγ* gene in DR.
